# Tislelizumab plus chemotherapy followed by anlotinib maintenance therapy in a patient with extensive-stage small cell lung cancer and intracranial recurrence after palliative whole-brain radiotherapy: a case report and literature review

**DOI:** 10.3389/fimmu.2026.1937771

**Published:** 2026-09-09

**Authors:** Rong Ji, Xialin Chen, Juan Lang, Zhongkui Xiong

**Affiliations:** 1 Department of Radiation Oncology, Shaoxing Second Hospital, Shaoxing, Zhejiang, China; 2 School of Medicine, Shaoxing University, Shaoxing, Zhejiang, China; 3 Department of Oncology, Shaoxing People’s Hospital, Shaoxing, Zhejiang, China; 4 Department of Pathology, Shaoxing People’s Hospital, Shaoxing, Zhejiang, China

**Keywords:** brain metastases, chemotherapy, extensive-stage small cell lung cancer, immunotherapy, radiation therapy, tislelizumab

## Abstract

Small cell lung cancer (SCLC) accounts for approximately 15% of all lung cancer cases. It is characterized by an exceptionally high proliferative rate, a strong propensity for early metastasis, and a poor prognosis. The median overall survival (OS) following brain metastases development in extensive-stage SCLC (ES-SCLC) was 10.5 months. Herein, a 74-year-old Chinese male was initially diagnosed with ES-SCLC with synchronous hepatic metastases. Following four cycles of first-line chemotherapy comprising etoposide and carboplatin, the patient underwent thoracic radiotherapy (TRT); however, prophylactic cranial irradiation was not performed. Brain metastases were detected 19 months after TRT, prompting initiation of palliative whole-brain RT (WBRT). Intracranial disease recurrence was observed five months after completion of WBRT. Managing intracranial recurrence following palliative WBRT in patients with ES-SCLC is clinically challenging. In this case of intracranial recurrence following palliative WBRT, the patient received second-line therapy comprising tislelizumab in combination with irinotecan and cisplatin chemotherapy, followed by anlotinib as maintenance therapy, achieving a complete response, sustained status of no evidence of disease for 24 months, and an OS of 67 months. Based on this case, tislelizumab combined with chemotherapy followed by anlotinib maintenance therapy represents a potentially viable treatment strategy for patients with recurrent intracranial lesions following palliative WBRT who have not received prior immune checkpoint inhibitor therapy. However, given the limited evidence derived solely from a single case, the clinical utility of this regimen requires validation in prospective and controlled trials.

## 1 Introduction

Small cell lung cancer (SCLC) accounts for approximately 15% of all lung cancer cases and is characterized by an exceptionally high proliferative rate, strong propensity for early metastasis, and poor prognosis (1). Approximately 70% of patients are diagnosed with extensive-stage SCLC (ES-SCLC) at initial presentation (2). A meta-analysis revealed that SCLC most commonly metastasizes to the liver (33%), brain (30%), bone (27%), adrenal glands (10%), and pericardium (3%) (3). Brain and liver metastases are poor prognostic factors in patients with ES-SCLC, as demonstrated in a retrospective cohort study (4). Liver metastasis emerged as the strongest adverse prognostic factor among patients with distant metastasis from SCLC in a retrospective analysis of data from the Surveillance, Epidemiology, and End Results database (5). Although the median overall survival (OS) following brain metastases was 10.5 months, the presence of liver or bone metastases was significantly associated with poorer survival outcomes (3).

In the randomized, double-blind, phase III KEYNOTE-604 trial, first-line pembrolizumab plus etoposide and platinum significantly improved progression-free survival (PFS) but did not significantly improve OS in patients with ES-SCLC. Adding durvalumab, atezolizumab, adebrelimab, serplulimab, tislelizumab, or toripalimab to platinum-based chemotherapy significantly improved OS in patients with ES-SCLC, as demonstrated in the phase 3 CASPIAN, IMpower133, CAPSTONE-1, ASTRUM-005, RATIONALE-312, and EXTENTORCH trials (6–11). A phase 3 randomized controlled trial in patients with ES-SCLC demonstrated a 2-year OS rate of 13% with thoracic radiotherapy (TRT) versus 3% without TRT, and a 6-month PFS rate of 24% with TRT versus 7% without TRT (12). A phase II trial evaluating aderbelimab in combination with chemotherapy followed by sequential TRT as first-line therapy for ES-SCLC reported a median OS of 21.4 months and a median PFS of 10.1 months (13). Radiation therapy plays an important role in treating patients with brain metastases from SCLC (14). Patients with brain metastases should receive whole-brain RT (WBRT) according to the American Radium Society’s Appropriate Use Criteria for ES-SCLC (15).

Managing intracranial recurrence following palliative WBRT in patients with ES-SCLC who have undergone sequential radiochemotherapy is clinically challenging. In this study, we report a case of intracranial recurrence following palliative WBRT for ES-SCLC, in which the patient achieved a complete response (CR) and prolonged survival with tislelizumab combined with chemotherapy, followed by anlotinib maintenance therapy.

## 2 Case presentation

**Figure 1:**
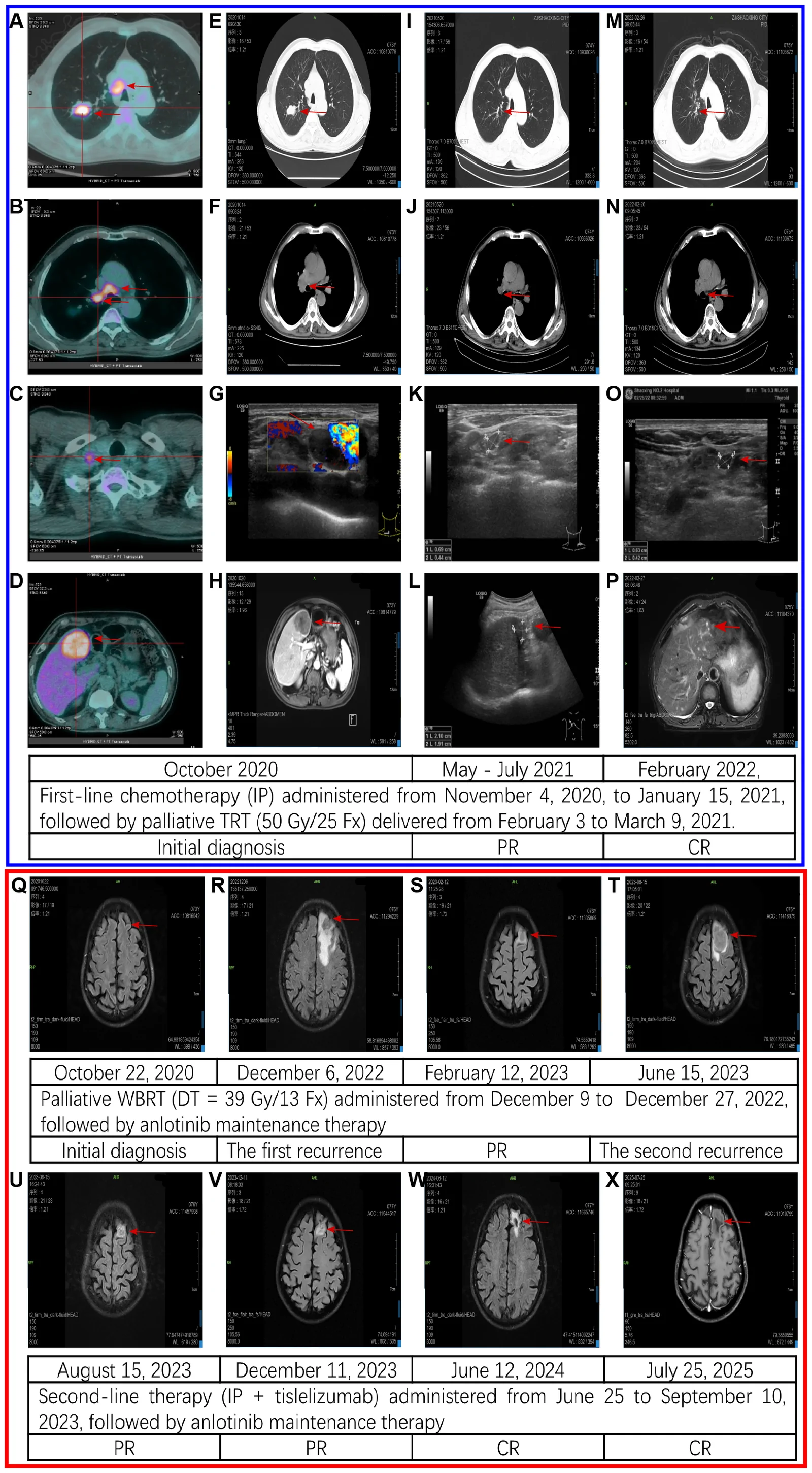
Imaging findings were evaluated during the diagnostic, therapeutic, and follow-up phases. Radiographic assessment during first-line systemic therapy and subsequent clinical follow-up **(A–P).** PET/CT scan performed at the time of initial diagnosis on October 23, 2020 **(A–D),** revealing a lobulated soft-tissue mass (33 × 25 mm) in the posterior segment of the right upper lobe, with markedly increased focal FDG uptake (SUVmax = 7.33), as indicated by the bottom-left arrow **(A)**, multiple mediastinal lymph nodes exhibited abnormally increased FDG uptake and confluent morphology, indicated by the top-right arrow **(A)**, a markedly hypermetabolic 20-mm lymph node was identified in the right hilar region (arrow, lower left; B), hypermetabolic mediastinal lymph nodes, indicated by the upper-right arrow **(B)**, a 12-mm right supraclavicular lymph node with markedly increased FDG uptake (SUVmax ≈ 4.4) **(C)**, and a 62-mm low-density soft-tissue mass identified in the medial segment of the left hepatic lobe with FDG uptake markedly increased (SUVmax ≈ 7.24) **(D)**. Imaging findings obtained at the time of initial diagnosis in October 2020 **(E–H)**. Chest CT revealed a 35 × 24 mm solid mass in the right upper lobe **(E)** and enlarged lymph nodes at the right hilum **(F)**. ultrasound exhibited a 17 × 10 mm enlarged right supraclavicular lymph node **(G)**. Abdominal contrast-enhanced MRI revealed a 55 × 52 mm mass in the medial segment of the left liver **(H)**. Response assessed by imaging from May to July 2021 was a PR **(I–L)**. A chest CT revealed no right upper lobe pulmonary mass **(I)** and no mediastinal or bilateral hilar lymphadenopathy **(J)**. Ultrasound images exhibited no pathologically enlarged lymph nodes in the right supraclavicular region **(K)**, and a 21 × 19 mm hyperechoic lesion in the left medial liver segment **(L)**. Response assessed by imaging on February 26–27, 2022, was a CR **(M–P)**. A chest CT revealed no pulmonary malignancy **(M)** or mediastinal/bilateral hilar lymphadenopathy **(N)**. Ultrasound images exhibited no pathologically enlarged right supraclavicular lymph nodes **(O)**. Hepatic MRI revealed no neoplastic lesions in the left lobe **(P)**. MR assessment of intracranial metastases **(Q–X)**. Brain MRI performed on October 22, 2020 (at initial diagnosis) exhibited no abnormal cerebral parenchymal signal **(Q)**. On December 6, 2022, a brain MRI revealed a 27 × 18 mm left frontal lobe lesion with abnormal signal, ill-defined margins, restricted diffusion, and perilesional edema **(R)**. These imaging features provided the first radiographic evidence of relapse. On February 12, 2023, a focal abnormal signal in the left frontal lobe was observed with slightly indistinct margins, ~11 × 12 mm **(S)**. Compared with the MRI from December 6, 2022, the lesion has decreased in size. Efficacy indicates PR. On June 15, 2023, a brain MRI exhibited a 27 × 31 mm left frontal lobe mass with an abnormal signal and slightly indistinct margins, larger than the lesion observed on February 12, 2023, confirming the second tumor relapse **(T)**. On August 15, 2023, the brain MRI exhibited a 19 × 18 mm nodular lesion in the left frontal lobe with abnormal signal **(U)**, reduced in size compared with the lesion on MRI imageon June 15, 2023. Efficacy evaluation indicates PR. Brain MRI performed on December 11, 2023, revealed a 12 × 10 mm nodular lesion with abnormal signal and indistinct margins in the left frontal lobe **(V)**. Compared with the MRI performed on August 15, 2023, the lesion decreased in size, consistent with PR. Brain MRIs dated June 12, 2024 **(W)** and July 25, 2025 **(X)** exhibited irregular, patchy liquefactive necrosis in the left superior frontal lobe, and the radiological assessment indicated a CR.

**Figure 2:**
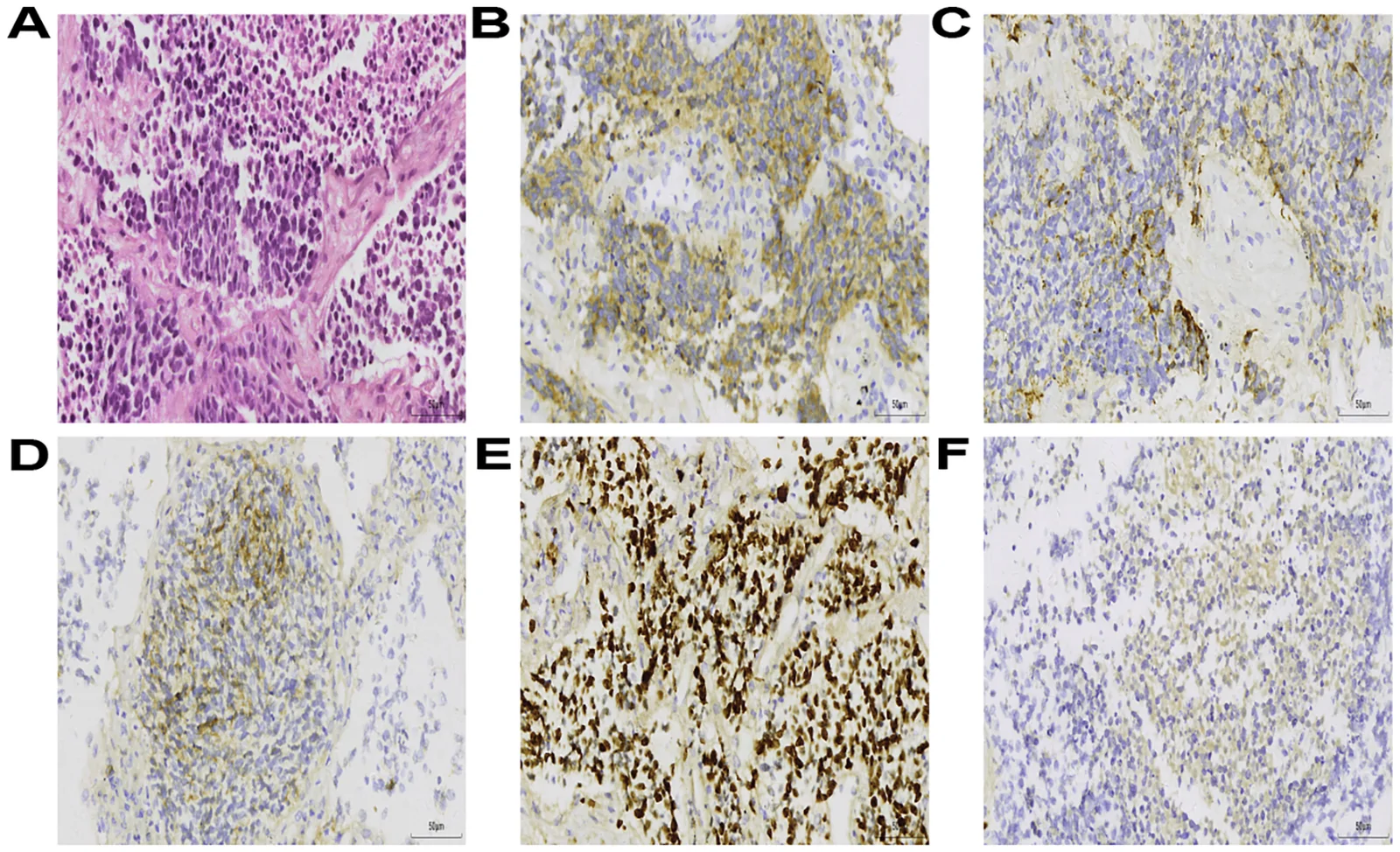
Pathological findings from the initial diagnostic lung biopsy specimens, visualized at ×400 magnification with a 50-μm scale bar. Hematoxylin and eosin staining **(A)**. Immunohistochemical staining **(B–F)**: Synaptophysin (Syn) **(B)**; Chromogranin A (CgA) **(C)**; CD56 **(D)**; Ki-67 **(E)**; thyroid transcription factor-1 (TTF-1) **(F)**.

A 74-year-old Chinese male was admitted to the Department of Thoracic Surgery, Shaoxing Second Hospital on October 19, 2020, for evaluation and management of a six-day history of suspected space-occupying lesions in the right lung and liver. Clinical assessment revealed no subjective symptoms, and physical examination was unremarkable. He reported no prior history of tobacco use or alcohol consumption. The patient had no documented history of hypertension, diabetes mellitus, chronic pulmonary disease, chronic liver disease, or occupational illness. The patient’s Eastern Cooperative Oncology Group Performance Status (ECOG PS) score was 0. On October 13, 2020, routine health screening at Qianqing Health Center, Shaoxing City revealed a right pulmonary mass on chest radiography and a hepatic mass on abdominal ultrasonography. The patient underwent a comprehensive diagnostic imaging workup between October 19 and 23, 2020, including neck and abdominal ultrasonography (US), contrast-enhanced chest computed tomography (CT), magnetic resonance imaging (MRI) of the upper abdomen and brain, and whole-body ^18^F-fluorodeoxyglucose positron emission tomography computed tomography (PET/CT) (**Figures 1A–H, Q**). An ultrasound-guided biopsy of the right supraclavicular lymph node was performed on October 26, 2020, and a CT-guided biopsy of the right upper-lobe pulmonary mass was performed on October 27, 2020 (**Table 1**; **Figures 2A–F**). The patient was initially diagnosed with SCLC at clinical stage IV (cT2N3M1) according to the eighth edition of the AJCC Cancer Staging Manual. The patient subsequently received four cycles of first-line chemotherapy, consisting of intravenous etoposide (100 mg, d1-3) and carboplatin (500 mg, d1), administered on day 1 of each 3-week cycle, from November 4, 2020, to January 15, 2021. Palliative TRT was delivered to visible thoracic lesions, including the primary pulmonary lesion and mediastinal lymph nodes, using 6-MV X-rays, with a total prescribed dose of 50 Gy delivered in 25 fractions (50 Gy/25Fx) from February 3 to March 9, 2021. The patient underwent cervical and upper abdominal US and chest CT imaging from May 19 to 20, 2021, to assess treatment response (**Figures 1I–L**). The response was assessed as a partial response (PR) according to RECIST 1.1 criteria. Chest CT, neck US, and upper abdominal MRI were performed on February 26–27, 2022, to assess treatment response (**Figures 1M–P**). The evaluation revealed a CR. The patient did not receive prophylactic cranial irradiation (PCI).

**Table 1:** Pathological results.

Date	Samples	Histopathological analysis	Immunohistochemistry analysis
October 31, 2020	The right supraclavicular lymph node biopsy	A poorly differentiated carcinoma with morphologic and immunohistochemical features consistent with SCLC.	CK7 (–), CK5/6 (–), p40 (–), TTF-1 (+), Napsin A (–), p53 (wild-type), Ki-67 (~80% +), CgA (focal +), Syn (weak +), CD56 (+).
November 3, 2020	The right upper lobe pulmonary mass biopsy	A malignant neoplasm consistent with SCLC.	CK7 (focal weak +), CK5/6 (−), p40 (−), TTF-1 (−), Napsin A (−), p53 (wild-type), Ki-67 (80% +), CgA (+), Syn (+), CD56 (+).

On December 6, 2022, follow-up cranial MRI revealed a brain metastasis measuring 27 × 18 mm in the left frontal lobe with surrounding vasogenic edema (**Figure 1R**). No positive neurological symptoms or signs were identified. These imaging features provided the first radiographic evidence of relapse. Palliative WBRT was administered on December 9, 2022, delivering a total dose of 30 Gy in 10 fractions (DT = 30 Gy/10 Fx). Subsequently, a boost of 9 Gy in 3 fractions was delivered to the brain metastases. The patient subsequently initiated anlotinib capsule therapy (12 mg orally once daily on days 1–14, repeated every 3 weeks). A follow-up brain MRI performed on February 12, 2023, revealed a reduction in the left frontal lobe metastasis (11 × 12 mm) (**Figure 1S**). The response was assessed as PR. On June 15, 2023, a brain MRI revealed a mass exhibiting abnormal signal intensity in the left frontal lobe, with slightly indistinct margins and approximate dimensions of 27 × 31 mm (**Figure 1T**, **Supplementary Figure S1**). This lesion was larger than the one identified on February 12, 2023. No positive neurological symptoms or signs were found.

Given evidence of a second relapse of brain metastases, second-line combination systemic therapy comprising an IP regimen (irinotecan 100 mg plus intravenous cisplatin 40 mg on days 1–3) plus intravenous tislelizumab 200 mg was initiated on June 25, 2023. Following the first cycle, the patient developed severe myelosuppression complicated by febrile neutropenia, which resolved after administering granulocyte colony-stimulating factor (G-CSF). The patient received two additional cycles of modified IP chemotherapy (irinotecan 100 mg + cisplatin 30 mg intravenously, on days 1–3) combined with tislelizumab 200 mg intravenously, on July 22 and August 15, 2023. A subsequent brain MRI performed on August 15, 2023, demonstrated a marked reduction in the size of the left frontal lobe metastasis, measuring 19 × 18 mm (**Figure 1U**). Compared with the February 12, 2023, scan, the response was assessed as PR. While pulmonary lesions remained stable, no residual tumor was detected at previously documented sites of liver and cervical lymph node metastases. On September 10, 2023, the patient received another cycle of intravenous irinotecan (100 mg) on days 1–3 plus intravenous tislelizumab (200 mg).

Tumor treatment efficacy and adverse events were assessed using laboratory tests. Neuron-specific enolase (NSE) peaked at 14.49 ng/mL on November 25, 2020, then declined rapidly, with transient elevations during the first and second disease recurrences. However, it remained below the upper limit of normal (**Supplementary Figure 2A**) Adverse events included grade 3 leukopenia (**Supplementary Figure 2B**), which improved following supportive treatment with G-CSF; grade 1 anemia (**Supplementary Figure 2C**); grade 1 thrombocytopenia (**Supplementary Figure 2D**); grade 1 elevated alanine aminotransferase (ALT) levels (**Supplementary Figure 2E**); grade 1 elevated aspartate (AST) levels (**Supplementary Figure 2F**), and grade 1 hypothyroidism (**Supplementary Figure 2G**).

**Figure 3:**
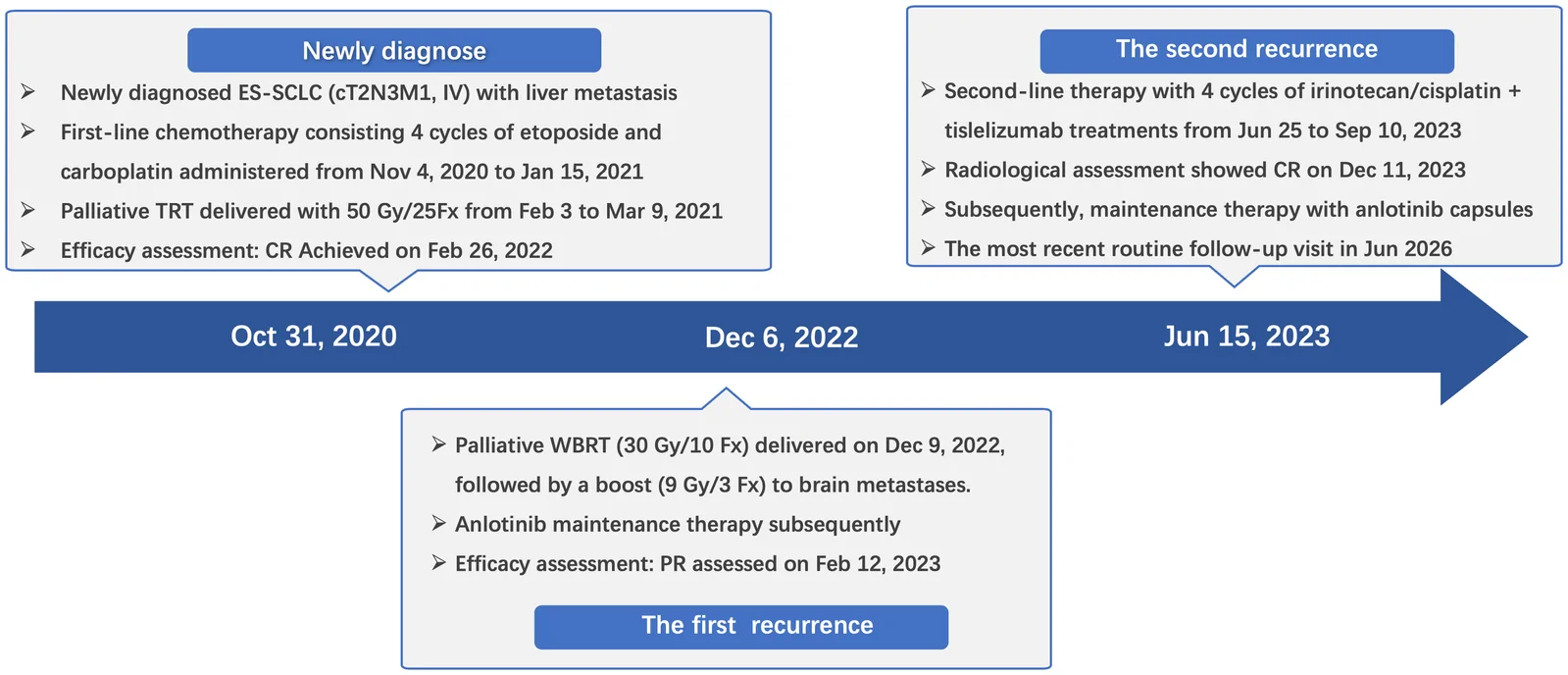
Clinical timeline for diagnosis and treatment.

Following second-line treatment, maintenance therapy was initiated with anlotinib capsules (12 mg administered orally once daily on days 1–14 of each 21-day cycle). The patient underwent regular surveillance. Brain MRI performed on December 11, 2023, revealed a nodular lesion measuring approximately 12 × 10 mm in the left frontal lobe (**Figure 1V**). Brain MRI performed on June 12, 2024 (**Figure 1W**) and July 25, 2025 (**Figure 1X**), revealed irregular, patchy areas of liquefactive necrosis in the left superior frontal lobe, consistent with post-treatment cystic degeneration. Radiological assessment indicated a CR. Cancer diagnosis timeline and treatment are presented in **Figure 3H**. At the most recent comprehensive follow-up visit, conducted on April 8, 2026, the patient remained in no evidence of disease (NED) for 24 months. At the most recent routine follow-up visit in June 2026, the patient remained alive with an ECOG PS score of 0 and an OS of 67 months.

## 3 Discussion

In this case, intracranial lesions recurred five months after palliative WBRT. A differential diagnosis between radiation-induced brain necrosis and tumor recurrence was clinically warranted. (I) On T2-weighted MRI sequences, radiation -induced brain necrosis typically manifests as a large, confluent area of hyperintensity with ill-defined margins (16). In contrast, recurrent metastatic tumors on T2-weighted imaging commonly exhibit isointense or mildly hyperintense signal intensity relative to normal brain parenchyma, with relatively well-demarcated borders (17). In this case, the patient exhibited isointense signal intensity relative to normal parenchyma on T2-weighted images, with focal areas demonstrating mild hyperintensity. (II) Radiation-induced brain necrosis typically evolves into the subacute phase 3–6 months after radiotherapy, during which necrotic tissue undergoes progressive liquefaction and disintegration, accompanied by a marked decrease in diffusion restriction (18). Diffusion restriction tends to be pronounced in malignant tumors (19). In this case, diffusion restriction was observed on diffusion-weighted MRI. However, other diagnostic modalities, including perfusion-weighted imaging (PWI), magnetic resonance spectroscopy (MRS), PET/CT, and image-guided needle biopsy, were not undertaken.

Despite receiving PCI, intracranial recurrence occurs in 12%–45% of patients with SCLC (20). Intracranial recurrence after initial cranial irradiation in patients with ES-SCLC frequently poses a significant therapeutic challenge, owing to the disease’s aggressive biology, its inherently poor overall prognosis, and concerns about cumulative neurotoxicity associated with re-irradiation (21). A retrospective study evaluating involved-field radiotherapy (IFRT) for recurrent brain metastases following PCI in patients with SCLC found that the IFRT group achieved significantly longer OS than the WBRT group across the entire cohort (17.80 vs. 8.47 months). However, no statistically significant difference in iPFS was observed between the IFRT and WBRT groups and no long-term radiotherapy-related toxicity was observed (22). The cumulative incidence of radiation necrosis has been reported to range from 5% to 12% following hypofractionated stereotactic radiosurgery delivering 27–35 Gy in 3–5 fractions (23).

Given the patient’s expressed preferences and values, and intracranial recurrence within six months following WBRT, re-irradiation was not administered; instead, the patient received four cycles of second-line systemic therapy comprising tislelizumab in combination with irinotecan and cisplatin, followed by maintenance therapy with anlotinib. In a six-study analysis of real-world treatment patterns, real-world OS in first-, second-, and third-line settings was consistently poor across countries, despite variations in patient characteristics and treatment practices (24). (I) Second-line chemotherapy in combination with ICIs, including a Phase I/II LUPER study evaluating lurbinectedin in combination with pembrolizumab for relapsed SCLC, the median PFS was 4.6 months, and the median OS was 10.5 months (25). In a real-world retrospective study, recurrent ES-SCLC receiving sintilimab in combination with chemotherapy in the second-line or later setting achieved a median PFS of 5.07 months, compared with 2.45 months for those receiving chemotherapy alone; median OS was 14.43 months versus 10.34 months, respectively (26). Second- or later-line serplulimab in combination with chemotherapy for SCLC demonstrated clinically meaningful benefits regardless of prior immunotherapy exposure, with a real-world analysis reporting a median OS of 12.00 months and a median PFS of 4.07 months (27). Based on the three aforementioned studies, the median PFS was 4.07–5.07 months for patients receiving immunotherapy in combination with chemotherapy, compared with 2.45 months for those receiving chemotherapy alone. (II) Second-line immunotherapy in combination with anti-vascular targeted therapy and chemotherapy, including tislelizumab, anlotinib, and irinotecan as second-line therapy for ES-SCLC post-platinum-based chemotherapy or ICIs in a single-arm, open-label, phase II trial; median PFS was 7.1 months, and median OS was 7.5 months (28). It is evident that the median PFS was extended to 7.1 months with the addition of anti-vascular targeted therapy to chemoimmunotherapy; however, the median OS did not show a corresponding improvement relative to historical literature. (III) Maintenance treatments, including atezolizumab continuation therapy, demonstrated promising efficacy and a favorable safety profile in patients with ES-SCLC who experienced disease progression following first-line chemoimmunotherapy, yielding a median PFS of 4.07 months and a median OS of 18.87 months (29). A single-arm, single-center, retrospective study evaluating anlotinib as maintenance therapy in patients with ES-SCLC reported a median PFS of 7.2 months and a median OS of 17.6 months (30). Based on the two aforementioned studies, maintenance therapy appears to confer a longer median OS. (IV) Comparative analysis of different second-line systemic therapies, including second-line therapy with PD-(L)1 inhibitors plus anlotinib yielded the longest median PFS (7.2 months) and median OS (33.5 months), significantly outperforming both PD-(L)1 inhibitors plus chemotherapy (median PFS: 4.6 months and median OS: 19.0 months) and chemotherapy alone (median PFS: 3.4 months and median OS: 18.3 months), as demonstrated in a retrospective analysis across three cancer centers (31). Topotecan is recognized as a standard second-line therapeutic option, with a median OS of 6 months (32). Given the patient’s second recurrence, localized exclusively to the intracranial compartment, occurring merely six months after completion of WBRT, the survival benefit conferred by second-line chemotherapy alone is likely limited. Based on the results of the aforementioned small, retrospective clinical study, we administered tislelizumab in combination with irinotecan and cisplatin as second-line therapy. This immunotherapy-based regimen shows promise for improving PFS. Subsequent maintenance therapy with anlotinib may further extend OS.

The patient attained a sustained tumor-free state following a therapeutic regimen comprising WBRT, subsequent treatment with tislelizumab in combination with chemotherapy, and maintenance therapy with anlotinib. SCLC exhibits poor response to immunotherapy (33). Although WBRT is not curative when administered after the detection of MRI-detectable tumors (34), radiation has been shown to downregulate claudin-5 expression and compromises blood-brain barrier (BBB) integrity (35). A blood–brain barrier–like vascular gate (BVG) in SCLC is distinct from NSCLC and other malignancies, characterized by tightly apposed endothelial cells, a thickened basement membrane, and dense pericyte coverage (33). Blood–tumor barrier (BTB) permeability is elevated in both low- and high-permeability brain metastases 2–4 weeks following radiotherapy (36). However, in another study, BBB permeability did not increase significantly until 90 days after fractionated radiation therapy, after which it rose continuously until 180 days post-fractionated radiation therapy (37). The patient had previously received WBRT, a treatment that may transiently increase BTB or BBB permeability and thereby open a subset of BVGs, facilitating intracranial delivery of the immune checkpoint inhibitors and chemotherapeutic agents to brain metastases. Given that the disease is characterized by a high recurrence rate (38), maintenance therapy appears to be clinically warranted. In a retrospective study of anlotinib as maintenance therapy after first-line chemotherapy plus consolidation radiation for ES-SCLC, 3-year OS was 27.6% with anlotinib versus 12.6% with observation (39). Consequently, the patient was initiated on maintenance targeted therapy with anlotinib following immunotherapy plus chemotherapy.

This case report has several inherent limitations: (I) The patient presented with synchronous liver metastases at initial diagnosis, but the liver metastases were not confirmed histopathologically; (II) neither the first nor the second brain metastasis was histologically confirmed by biopsy; (III) needle biopsies were obtained from the supraclavicular lymph nodes and lung, but neither sample underwent subsequent next-generation sequencing or molecular profiling; (IV) Several diagnostic modalities, including perfusion-weighted imaging, magnetic resonance spectroscopy, PET/CT, and image-guided needle biopsy, were not performed during the second recurrence, despite their utility in differentiating tumor recurrence from radiation-induced brain necrosis.

In this case of intracranial recurrence following palliative WBRT, second-line therapy with tislelizumab in combination with irinotecan and cisplatin chemotherapy, followed by anlotinib as maintenance therapy, achieved a CR, sustained status of NED for 24 months, and an OS of 67 months. These findings suggest that tislelizumab combined with chemotherapy followed by anlotinib maintenance therapy represents a potentially viable treatment strategy for patients with recurrent intracranial lesions following palliative WBRT, who had not received prior ICI therapy. However, given the limited evidence from a single case, the clinical utility of this regimen requires validation in prospective and controlled trials.

## Data Availability

The raw data supporting the conclusions of this article will be made available by the authors, without undue reservation.

## Supplementary material

Supplementary Figure 1 MR images. On June 15, 2023, a brain MRI exhibited a 27 × 31 mm left frontal lobe mass with an abnormal signal and slightly indistinct margins, larger than the lesion observed on February 12, 2023, confirming the second tumor relapse. T1-weighted image demonstrated signal intensity ranging from isointense to mildly hypointense relative to adjacent reference tissue **(A)**; T2-weighted image demonstrated signal intensity ranging from isointense to mildly hyperintense relative to adjacent normal tissue **(B)**; Diffusion restriction was observed on diffusion-weighted imaging **(C)**.

Supplementary Figure 2 Laboratory test results. In the figures, the x-axis represents the date, and the y-axis represents the concentration values obtained from laboratory assays. The core needle biopsy of the right supraclavicular lymph node date (October 31, 2020) was designated as the baseline reference time point (day 0). In these figures, the two red horizontal dashed lines indicate the upper and lower reference limits of the laboratory test results. The blue vertical dashed line denotes the first tumor recurrence date (December 6, 2022), and the purple vertical dashed line denotes the second tumor recurrence date (June 15, 2023). NSE **(A)**; White blood cell (WBC) count **(B)**; Hemoglobin (HB) **(C)**; Platelet (PLT) count **(D)**; ALT **(E)**; AST **(F)**; total triiodothyronine (TT3) **(G)**.
