# Supplementary material for: Tislelizumab plus chemotherapy followed by anlotinib maintenance therapy in a patient with extensive-stage small cell lung cancer and intracranial recurrence after palliative whole-brain radiotherapy: a case report and literature review
Source: Front Immunol. 2026 Sep 9;17:1937771. doi: 10.3389/fimmu.2026.1937771 (PMC13597218; doi:10.3389/fimmu.2026.1937771)
Supplement: Supplementary file 1 [file Table1.docx]

Table S1. Second-line systemic therapy for patients with recurrent SCLC and maintenance therapy in extensive-stage disease

|  | Study | Grouping | Efficacy | Treatment-related adverse events (TRAEs) | Authors | Reference |
| --- | --- | --- | --- | --- | --- | --- |
| Second-line chemotherapy in combination with ICIs | Phase I/II LUPER stud evaluating lurbinectedin in combination with pembrolizumab for relapsed SCLC | Lurbinectedin in combination with pembrolizumab (n = 28) | Median PFS was 4.6 months, and the median OS was 10.5 months | Grade 3+TRAEs occurred in 71.4% of patients, most commonly transient neutropenia; irAEs were consistent with prior pembrolizumab studies. | Calles A, et al. | 25 |
|  | Real-world retrospective study on recurrent ES-SCLC receiving sintilimab in combination with chemotherapy | Sintilimab in combination with chemotherapy (n = 24) versus chemotherapy alone (n = 22) | Median PFS: 5.07 months versus 2.45 months; median OS: 14.43 months versus 10.34 months | In the sintilimab/chemotherapy and chemotherapy groups, TRAEs occurred in 18 (75.0%) and 14 (63.6%) patients, and grade ≥3 TRAEs in 3/24 (12.5%) and 4/22 (18.2%), respectively. | Wang J, et al. | 26 |
|  | Real-world analysis of immunochemotherapy in recurrent SCLC | Serplulimab-based immunochemotherapy (n = 39) | Median PFS: 4.07 months; median OS: 12.00 months | Overall AE incidence: 61.54%; grade 1–2 AEs: 46.15%; grade ≥3 AEs: 5.13%. Most common AEs: thrombocytopenia, elevated ALT, and hypothyroidism. | Guo Y, et al. | 27 |
| Second-line immunotherapy in combination with anti-vascular targeted therapy and chemotherapy | Single-arm, open-label, phase II trial evaluating second-line therapy for ES-SCLC post-platinum-based chemotherapy or ICIs | Tislelizumab, anlotinib, and irinotecan (n = 39) | Median PFS was 7.1 months, and median OS was 7.5 months | Overall AE incidence: 61.54%; grade 1–2 AEs: 46.15%; grade ≥3 AEs: 5.13%. Most common AEs: thrombocytopenia, elevated ALT, and hypothyroidism. | Chen Y, et al. | 28 |
| Maintenance treatments | ES-SCLC who experienced disease progression following first-line chemoimmunotherapy | Atezolizumab continuation therapy (n =28) | Median PFS of 4.07 months and median OS of 18.87 months | Respiratory AEs were most common: cough, dyspnea, and pneumonitis (all 35.7%). Thyroiditis (17.9%) was the most frequent irAE. | Shi W, et al. | 29 |
|  | Single-arm, single-center, retrospective study evaluating anlotinib as maintenance therapy in patients with ES-SCLC | Anlotinib maintenance therapy (n = 30) | Median PFS and OS: 7.2 and 17.6 months, respectively | Grade 3–4 adverse events occurred in 10 patients (33.3%); no treatment-related deaths occurred. | Xiong J, et al. | 30 |
| Comparative analysis of different second-line systemic therapies | A retrospective analysis across three cancer centers | Second-line therapy with PD-(L)1 inhibitors plus anlotinib (n = 86), PD-(L)1 inhibitors plus chemotherapy (n = 125), chemotherapy alone (n = 178). | PD-(L)1 inhibitors plus anlotinib: (median PFS 7.2 months and median OS 33.5 months); PD-(L)1 inhibitors plus chemotherapy (median PFS: 4.6 months and median OS: 19.0 months); chemotherapy alone (median PFS: 3.4 months and median OS: 18.3 months), | The I+A group had the lowest incidence of ≥grade 3 myelosuppression versus I+C and C (3.5% vs. 11.2% vs. 16.3%; P < 0.01). However, I+A was associated with higher rates of immune-related pneumonia (2.3% vs. 0% vs. 0%; P < 0.05) and hemoptysis (1.4% vs. 0% vs. 0.6%; P < 0.05) than I+C and C. | Zhang J, et al. | 31 |

AEs: adverse events; irAE: immune-related adverse events; I + A: PD-(L)1 inhibitors plus anlotinib; I + C: PD-(L)1 inhibitors plus chemotherapy; C: chemotherapy alone.
